# Clinical, neuropsychological, and pre-stimulus dorsomedial thalamic nucleus electrophysiological data in deep brain stimulation patients

**DOI:** 10.1016/j.dib.2016.06.008

**Published:** 2016-06-15

**Authors:** Catherine M. Sweeney-Reed, Tino Zaehle, Jürgen Voges, Friedhelm C. Schmitt, Lars Buentjen, Klaus Kopitzki, Alan Richardson-Klavehn, Hermann Hinrichs, Hans-Jochen Heinze, Robert T. Knight, Michael D. Rugg

**Affiliations:** a Departments of Neurology and Stereotactic Neurosurgery, Otto von Guericke University, Leipziger Strasse 44, 39120 Magdeburg, Germany; b Department of Behavioral Neurology, Leibniz Institute for Neurobiology, Otto von Guericke University, Leipziger Strasse 44, 39120 Magdeburg, Germany; 3 German Centre for Neurodegenerative Diseases (DZNE), Otto von Guericke University, Leipziger Strasse 44, 39120 Magdeburg, Germany; d Helen Wills Neuroscience Institute and Department of Psychology, University of California, Tolman Hall, MC 3192, Berkeley, California 94720, USA; e Center for Vital Longevity and School of Behavioral and Brain Sciences, University of Texas, Dallas, TX 75235, USA

**Keywords:** Memory encoding, Dorsomedial thalamic nucleus, Pre-stimulus theta

## Abstract

The data presented here comprise clinical, neuropsychological, and intrathalamic electrophysiological data from 7 patients with pharmacoresistant focal epilepsy and are related to the article “Pre-stimulus thalamic theta power predicts human memory formation” C.M. Sweeney-Reed, T. Zaehle, J. Voges, F.C. Schmitt, L. Buentjen, K. Kopitzki, et al. (2016) [1]. The patients participated in a memory paradigm after receiving electrodes implanted in the DMTN due to the surgical approach taken in electrode insertion for deep brain stimulation of the anterior thalamic nucleus. Epilepsy duration and pre-operative neuropsychological tests provide an indication of the profile of patients receiving intrathalamic electrode implantation and the memory capabilities in such a patient group. The electrophysiological data were recorded from the right DMTN preceding stimulus presentation during intentional memory encoding. The patients viewed a series of photographic scenes, which they judged as indoors or outdoors. The 900 ms epochs prior to stimulus presentation were labeled as preceding successful or unsuccessful subsequent memory formation according to a subsequent memory test for the items. The difference between theta power preceding successful versus unsuccessful subsequent memory formation is shown against time for each patient individually.

**Specifications Table**

**Table:** 

Subject area	*Cognitive neuroscience*
More specific subject area	*Memory encoding, dorsomedial thalamic nucleus, pre-stimulus theta*
Type of data	*Table, figure*
How data were acquired	*Duration of epilepsy according to time from clinical diagnosis, reached following standard neurological assessment, until electrode implantation. Neuropsychological testing. Continuous electrophysiological data were recorded from two 1.5 mm platinum intrathalamic electrodes located in the right dorsomedial thalamic nucleus using a Walter Graphtek amplifier at a sampling frequency of 512 Hz against a nose reference, re-referenced to a bipolar montage, epoched 900 ms pre-stimulus to stimulus onset, labeled as preceding successful or unsuccessful memory formation, wavelet-transformed using a 6 cycle Morlet wavelet, and averaged across successful and unsuccessful encoding epochs. The difference between theta power preceding successful and unsuccessful encoding was calculated.*
Data format	*Analyzed*
Experimental factors	*Participants viewed 200 photographic scenes during electrophysiological data recording, judging them as indoors or outdoors. A subsequent memory test, in which the scenes were presented in a random order, intermixed with 100 new scenes, was used to label the memory encoding epochs as preceding successful or unsuccessful encoding.*
Experimental features	*N=7 participants*
Data source location	*Magdeburg, Germany*
Data accessibility	*Data are provided in this article*

**Value of the data**

• Clinical and neuropsychological data are provided from 7 patients with pharmacoresistant focal epilepsy who underwent intrathalamic electrode implantation for deep brain stimulation treatment. These data can inform researchers about the epilepsy duration and the possible neuropsychological profile of such patients, from whom intrathalamic electrophysiological recordings are used to provide insights into the mechanisms underlying cognition.
• The difference between pre-stimulus power in the dorsomedial thalamic nucleus (DMTN) preceding successful compared with unsuccessful memory formation is shown against frequency and time for the same 7 patients. Lesion and imaging evidence suggest a role for the DMTN in memory processing [2], [3], [4], and pre-stimulus hippocampal theta (4–8 Hz) power predicts memory formation [5]. These data could be compared with other recordings from this thalamic nucleus as well as from other brain structures during memory formation.
• The timing of the enhanced theta power preceding successful compared with unsuccessful encoding could be compared with data recorded from other brain structures during a similar memory encoding paradigm, or with data also recorded from the DMTN but using a different cognitive paradigm.

## 1 Data

**Fig. 1:**
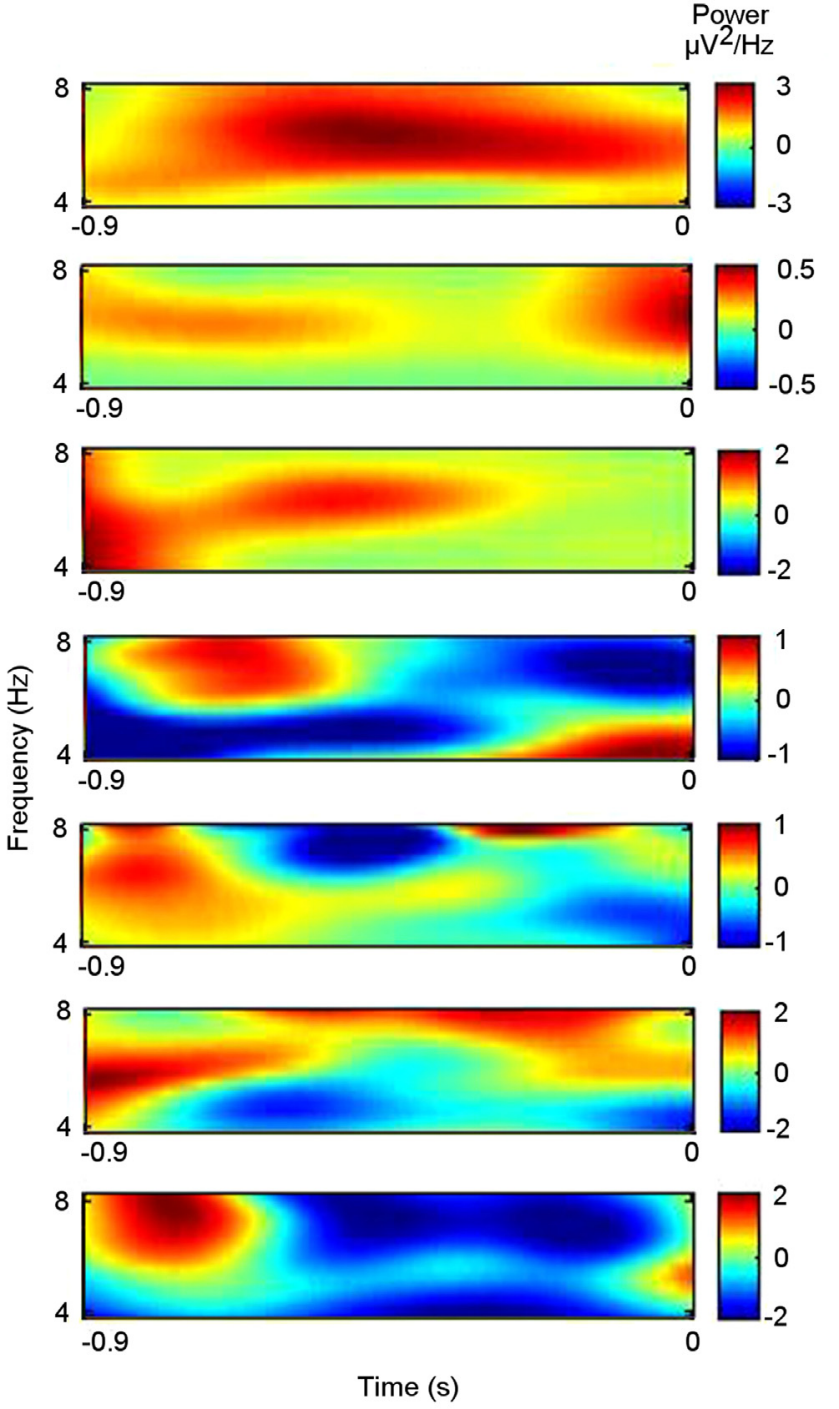
Difference between theta power preceding successful and unsuccessful memory formation in the right dorsomedial thalamic nucleus. The power difference is shown for 7 individual participants. (Note that the frequency is plotted according to a binary logarithmic scale.)

The duration of epilepsy and the pre-operative neuropsychological profile for the 7 patients are shown in Table 1. The difference between the right dorsomedial thalamic nucleus pre-stimulus theta (4–8 Hz) power preceding successful versus unsuccessful memory formation is shown over time for the 7 individual patients in Fig. 1.

## 2 Experimental design, materials and methods

The 7 participants received intrathalamic electrodes implanted for deep brain stimulation to treat pharmacoresistant focal epilepsy. The duration of epilepsy is provided for each patient in Table 1. Neuropsychological testing was performed prior to surgery, and the neuropsychological data are also shown in Table 1. The tests included the Hamburg–Wechsler Intelligence Test for Adults for IQ, verbal (VLMT) [6] and figural memory [7], and sustained attention [8]. The VLMT involves verbal presentation of word lists, followed by immediate and delayed recall and recognition. The figural memory test assesses visual memory through recall and reproduction of symmetrical geometrical drawings. Sustained attention was evaluated through application of a timed task, which participants were asked to carry out as quickly and accurately as possible. Rows of d׳s and p׳s were shown, with up to two marks above and up to two marks below. The goal was to eliminate the d׳s with exactly two marks at the top or two at the bottom, while ignoring other letters.

The electrophysiological data presented here were recorded from two electrode contacts located in the right dorsomedial thalamic nucleus (DMTN) of each patient. The anterior thalamic nucleus was the stereotactic target structure for stimulation, and contacts were additionally located in the DMTN due to deeper insertion of the electrode probe to provide stability. Electrode location was planned pre-operatively on the basis of structural MRI. Electrode placement was verified post-operatively by co-registering the pre-operative MRI scans with intraoperative X-ray images and post-operative CT, using comparison with standard atlas location of the DTMN [1], [9]. Details of electrode localization are available in [1]. The electrophysiological data presented here were recorded in the first few days post-operatively, before commencement of stimulation therapy. Data recording was approved by the Local Ethics Committee of the Otto-von-Guericke University Magdeburg, and all patients provided written, informed consent.

During right DMTN data recording, patients viewed a series of 200 photographic scenes, which they judged as indoors or outdoors by button press using left and right index fingers, counterbalanced across participants. The same scenes were subsequently shown again, in a random order, intermixed with 100 new scenes, and the patients were asked to judge whether the scenes had been shown previously.

The electrophysiological data were recorded at a sampling frequency of 512 Hz using a Walter Graphtek amplifier against a nose reference. They were re-referenced to a bipolar montage and epoched from 900 ms before the stimulus to the stimulus onset time point, which was marked as 0 s. The epochs were labeled as preceding successful or unsuccessful memory encoding according to a subsequent memory test, in which the 200 scenes were presented again in a random order, intermixed with 100 new scenes [10]. A time-frequency decomposition was performed using wavelet transformation with a 6 cycle Morlet wavelet, from which power was derived in the theta (4–8 Hz) frequency range [1]. The theta power was averaged across epochs preceding successful and preceding unsuccessful encoding, and the difference was determined and plotted against frequency and time for each participant.

## Figures and Tables

**Table 1:** Epilepsy duration and neuropsychological profile. HAWIE-R = Hamburg–Wechsler Intelligence Test for Adults. Z-scores are against age-appropriate norms.

Pt	Epilepsy duration (years)	IQ (HAWIE-R)	Verbal memory			Figural memory (z-score)	Sustained attention (z-score)
			Immediate free recall (z-score)	Delayed free recall (z-score)	Recognition (z-score)		
1	19	95	−1.6	−1.6	−1.6	−2	0.2
2	31	91	−1.3	−0.8	−1.3	−1	−0.5
3	9	124.9	−0.65	−0.2	−1.1	−1	0
4	12	94	−0.4	−0.2	−1.1	−1	−0.8
5	19	74.2	−0.65	−1.6	−1	−1.6	−2.2
6	12	103	−0.1	−0.9	−0.5	−0.8	0.7
7	30	96	0.3	0.3	−0.1	0.8	0.5
